# Shifting perceptions: a pre-post study to assess the impact of a senior resident rotation bundle

**DOI:** 10.1186/1472-6920-13-115

**Published:** 2013-08-29

**Authors:** Gabriel Fabreau, Meghan Elliott, Suneil Khanna, Evan Minty, Jean E Wallace, Jill de Grood, Adriane Lewin, Garielle Brown, Aleem Bharwani, Janet Gilmour, Jane B Lemaire

**Affiliations:** 1 Faculty of Medicine, Health Sciences Centre, Foothills Campus, University of Calgary, 3330 Hospital Drive NW, Calgary AB T2N 4N1, Canada; 2 University of Calgary, 2500 University Dr NW, Calgary AB T2N 1N4, Canada; 3 W21C Research and Innovation Center, GD01 TRW Building, 3280 Hospital Drive, NW, Calgary AB T2N 4Z6, Canada

## Abstract

**Background:**

Extended duty hours for residents are associated with negative consequences. Strategies to accommodate duty hour restrictions may also have unintended impacts. To eliminate extended duty hours and potentially lessen these impacts, we developed a senior resident rotation bundle that integrates a night float system, educational sessions on sleep hygiene, an electronic handover tool, and a simulation-based medical education curriculum. The aim of this study was to assess internal medicine residents’ perceptions of the impact of the bundle on three domains: the senior residents’ wellness, ability to deliver quality health care, and medical education experience.

**Methods:**

This prospective study compared eligible residents’ experiences (N = 67) before and after a six-month trial of the bundle at a training program in western Canada. Data was collected using an on-line survey. Pre- and post-intervention scores for the final sample (N = 50) were presented as means and compared using the t-test for paired samples.

**Results:**

Participants felt that most aspects of the three domains were unaffected by the introduction of the bundle. Four improved and two worsened perception shifts emerged post-intervention: less exposure to personal harm, reduced potential for medical error, more successful teaching, fewer disruptions to other rotations, increased conflicting role demands and less staff physician supervision.

**Conclusions:**

The rotation bundle integrates components that potentially ease some of the perceived negative consequences of night float rotations and duty hour restrictions. Future areas of study should include objective measures of the three domains to validate our study participants’ perceptions.

## Background

Extended duty hours for resident physicians have been associated with negative consequences such as decreased working memory, worsening cognitive performance and increased technical error [1-8], poor performance on learning measures, patient safety concerns, increased workplace injury, burnout, and personal harm [1-4,6,7,9-19]. Restriction of consecutive duty hours has been proposed as a means to reduce these negative consequences.

Various scheduling strategies have been used to accommodate duty-hour restrictions for residents. One approach is the night float rotation system, whereby residents provide patient care either during daytime shifts or during 12 to 16 hour overnight shifts [3,9,10,12,16]. Despite research showing the benefits of reduced consecutive duty hours and night float rotations, some contradictory evidence continues to emerge about the impact on residents’ wellness, ability to deliver health care and medical education experience [9,12,14,20-22]. Night float rotations can cause disruptions in basic biological functions, circadian rhythms, social relationships, and psycho-physical health [23]. The literature suggests that sleep hygiene strategies may help [23]. Shorter work shifts necessitate more frequent handover of patient care responsibilities between house staff creating the potential for lapses in continuity of care and communication [24]. The aviation, nuclear power generation, military, and space exploration industries have shown a reduction in communication errors through a standardized approach to transfer of responsibility and the use of handover tools [25-27]. Electronic health care communication tools have been associated with improved transfer of information amongst house staff and attending physicians, increased handover efficiency and decreased communication errors [28,29]. Medical education research related to restricted duty hours has revealed contradictory results with some studies showing no change and others an improvement in overall quality [9,12,14,30] but with raised concerns over the potential diminution in overall exposure to the number and variety of patient cases [11,14,16]. Simulation training, defined as a person, device, or set of conditions which attempts to present medical problems authentically for the purpose of education or evaluation [31], is an increasingly popular and effective medical education innovation [7,31-34].

In 2010, the chief internal medicine residents at our institution designed a new senior resident rotation bundle. This innovative program was intended to eliminate extended duty hours for internal medicine senior residents and to address some of the identified consequences of duty hour restrictions on residents’ wellness, health care delivery and medical education experience. The SRRB integrated three components: 1) a night float system with supplementary educational sessions about sleep hygiene and circadian rhythm changes; 2) an electronic patient-care handover communication tool with supplementary educational sessions about tool use, effective handover of patient care, and critical incident experiences in other industries; and 3) a supplemental simulation-based educational curriculum.

The aim of this study was to measure a cohort of internal medicine residents’ perceptions of the senior residents’ wellness, quality of health care delivery, and medical education experience before and after a 6-month pilot implementation of the senior resident rotation bundle (SRRB).

## Methods

### Study design, setting, and participants

This prospective pre-post study compared a cohort of internal medicine residents’ attitudes and experiences before and after a six-month pilot implementation of the SRRB. The intervention was implemented at two large academic hospitals within an internal medicine residency training program in western Canada. Eligible participants were all internal medicine residents from the core program (N = 67) including the junior residents (post-graduate year 1), and the senior residents (post-graduate year 2 and 3). All residents were deliberately included in the study cohort for several reasons. During the planning stages of the project, there was voiced uncertainty regarding the acceptance of a permanent change in the senior resident rotation schedule from the senior residents who were concerned about the immediate impact, and from the junior residents given their upcoming inevitable transition into the senior role. Also, the measured aspects of the senior residents’ self and work (wellness, ability to deliver quality health care, medical education experience) all have the potential to affect both junior and senior resident colleagues either directly (e.g. allowing healthy relationships, teaching successfully) or indirectly (e.g. achieving general wellness, experiencing rotation disruptions) thus all residents’ perceptions of the intervention’s benefits and harms for the senior were of interest. Data were collected using an on-line survey. Recruitment was by e-mail and included a message from the principal investigator on behalf of the research team and a link to the on-line survey. Pre-intervention surveys were sent out by e-mail on August 27th, 2010, followed by reminders on September 1st and 24th. The pilot intervention was implemented on August 30th, and ran until February 14th, 2011. Post-intervention surveys were sent out on February 28th, followed by reminders on March 7th and 18th. Only those who responded to both the pre- and post-intervention surveys were included in the paired data analysis. Respondents were anonymized and pairing was achieved through assigning unique identifiers for each resident. This study received ethics approval from the Conjoint Health Research Ethics Review Board at the University of Calgary.

### Intervention

Prior to the 6 month pilot, there were two senior resident roles on the Medical Teaching Unit (MTU) at our institution: the ward senior, who does not participate in night call during the week, and the emergency liaison senior who works 26 hour on-call shifts up to 7 times per month. Senior residents from non-MTU rotations were also required to fulfill some overnight MTU call shifts, resulting in post-call absences from their assigned rotations. The SRRB, which replaced this scheduling system, consisted of three components. First, a night float rotation was established, where one senior resident works five consecutive weeknights from 2000-0900 hours with a complementary bridge shift staffed by other senior residents from 1700-2000 hours. Residents were offered supplemental educational sessions about sleep hygiene and circadian rhythm changes prior to the pilot period. Second, a new patient care handover communication tool was designed and integrated into the existing electronic patient care information system, to replace the existing in-person hand-written handover method. The tool auto-populates patient demographic information, and allows for text entry of a patient’s medical profile, ongoing medical issues and care plans. Daily updates are then provided by residents and medical students. Supplemental educational sessions were offered to the residents in our study outlining how to use the tool, the importance of thorough face-to-face handover, and examples of critical incident experiences in other non-health care industries. Residents were taught to apply specific communication components when updating the handover tool such as highlighting patients that may require a higher degree of overnight attention due to severity or complexity of illness and documenting a concise report of current medical problems, treatment plans, and follow-up issues, with specific instructions on how and when to complete these tasks. Residents were provided iterative feedback on the quality and completeness of their handover by staff and fellow residents on an ongoing basis to ensure improved quality of communication and compliance with the training provided. Third, an education week focused on a simulation-based learning and teaching curriculum for the senior residents and free of patient care duties was incorporated into the 4 week MTU rotation. The senior residents created high fidelity learning scenarios, and/or used simulation equipment to practice medical procedures (e.g. paracentesis, thoracentesis, central line insertion, lumbar puncture, knee arthrocentesis, and/or fundoscopy). These skills were then taught to junior residents and medical students on the MTU. These simulation activities were split amongst the two participating hospitals. Internal medicine faculty and clinical preceptors strongly encouraged adherence to duty hour reductions ensuring senior residents left the hospitals promptly upon completion of an overnight shift.

### Outcomes and survey instrument

The primary outcome was the change in all internal medicine residents’ perceptions of senior residents’ wellness, ability to deliver quality health care and medical education experience, pre- and post-intervention, as measured by a questionnaire. The pre- and post-surveys were constructed by the research team to measure outcomes of interest and themes identified in the relevant literature. Wherever possible, validated items or scales were used to measure variables of interest. Where there was little to no existing research in the field, we consulted previous physician wellness research [35] and key stakeholders including residents, staff physicians, and nurses from the MTU to develop new items. For example, new items were developed to measure aspects specific to our program, the simulation curriculum and the rotation scheduling. Likert items measured the extent to which respondents agreed or disagreed with statements relating to the three major domains of the senior residents’ wellness (16 items), ability to deliver quality health care (17 items), and medical education experience (16 items) (Table 1). The responses to each item were coded as strongly disagree (1), disagree (2), neither agree nor disagree (3) agree (4) and strongly agree (5).

**Table 1:** Study outcomes, scales, single items grouped into scales (R indicates reverse coding of item)

**Impact of Senior Resident Schedule on Seniors’ Wellness**	
**Allows general wellness**	Adversely affects their health (R); Restricts their participation in physical activity after work (R); Impairs their ability to adapt to Circadian Rhythm changes (R); Contributes to their overall sleep debt (R); Contributes to their overall fatigue levels (R); Contributes to frequent episodes of physical illness (e.g. colds) (R); Enhances their overall energy levels; Contributes to their need to use stimulants such as caffeine (R).
**Allows exposure to personal harm**	Impairs safety while driving home post call; Allows potential for workplace harm such as needle-stick injuries.
**Causes conflicting role demands**	Makes it easy for them to trade on call shifts with others (R); Allows free time to accomplish their non-work related errands (R); Provides opportunities to spend time with their family (R); Restricts their time available to do research.
**Allows healthy relationships**	Allows healthy interpersonal relationships.
**Causes feelings of isolation**	Causes them to feel isolated at times (R).
**Impact of Senior Resident Schedule on Seniors’ Ability to Deliver Quality Health Care**	
**Allows potential for error**	They are alert during procedures (R); They commit preventable medical errors; They experience "near misses" related to poor patient care; They are often too tired to provide safe patient care.
**Allows clinical skills expertise**	They miss important diagnoses (R); They manage complex medical patients appropriately; The content of their patient care handover is accurate; They perform a thorough work up of new admissions.
**Allows continuity of patient care**	They highlight important follow up items during handover of patient care issues; They maintain continuity of patient care; They assume accountability for the patients they admit.
**Causes expenditure of emotional labour**	Their interactions with other MTU team members are positive (R); They communicate well with patients and their families (R); They are sensitive to social issues pertaining to patient care (e.g. gender and culture) (R).
**Allows work efficiency**	They are able to effectively multitask during busy work times; They handover patient care issues in a time efficient manner; They respond to pages in a timely fashion.
**Impact of Senior Resident Schedule on Seniors’ Medical Education Experience**	
**Allows successful teaching**	They have enough time to teach junior residents and clerks; They have enough energy to teach junior residents and clerks; They are confident in their ability to teach procedural skills; They are confident in their ability to teach how to run a code; They are confident in their ability to teach how to manage unstable critically ill patients.
**Allows medical skills proficiency**	They are confident in their ability to perform procedures; They are confident in their ability to run a code; They are confident in their ability to manage unstable critically ill patients.
**Allows successful learning**	They have opportunities to learn procedures through simulation training; They can acquire new knowledge; They can retain new knowledge to apply to patient care; Their overall educational experience is satisfying.
**Allows staff physician supervision**	They have the opportunity to review cases with attending physicians; Their clinical skills (history and physical) are observed by an attending physician; They receive feedback from attending physicians.
**Causes rotation disruptions**	Their ambulatory care rotations are frequently interrupted due to MTU on call duties.

### Statistical methods

The individual Likert items were grouped into 15 scales, 5 for each domain (Table 1). Following reverse coding of individual items where appropriate, confirmatory factor analysis and reliability analysis were performed to determine that individual items in each of the 15 pre-specified scales loaded on a single factor (Table 1 and more detailed results available from authors). Scale scores were calculated by summing the individual Likert item scores and dividing by the number of items for a standardized scale score between 1 and 5. Respondents who completed both the pre- and post-surveys were included in this analysis. Differences in individual scale scores for pre-versus post-intervention surveys were assessed for normality using the Shapiro-Wilk test, and pre- and post-intervention scale scores were presented as means and compared using the t-test for paired samples. Given the multiple comparisons made under each of the three major outcome domains (senior resident wellness, ability to deliver quality health care and medical education experience) a p-value of <0.01 was used to indicate a statistically significant difference between pre- and post-scores. Data analysis was conducted using Stata version 11 (StataCorp LP, College Station, Texas).

**Figure 1:**
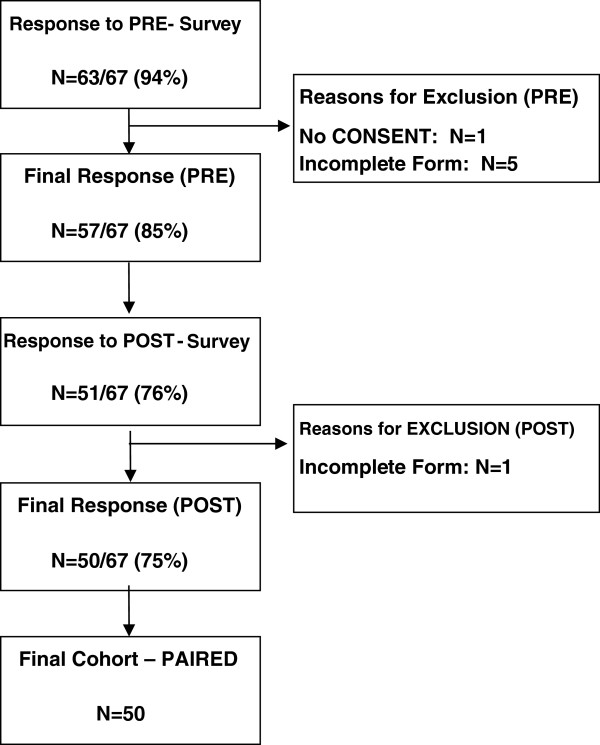
Study response and final sample.

## Results

### Participants

Sixty-three of 67 residents (94%) responded to the pre-intervention survey, with 57/67 (85%) eligible for study inclusion. Fifty-one of 67 residents responded to the post-intervention survey (76%), with 50/67 (75%) eligible for study inclusion. Reasons for exclusion included lack of documented consent (N = 1) and incomplete survey data (N = 6). Fifty residents formed the final paired cohort from which data were analyzed for this study (Figure 1). The mean age of the participants was 31 years (SD 6) and 23 (46%) were male. Thirty (60%) were married and 18 (36%) were single. Thirty (60%) declared a partner who works full-time for pay and 4 (8%) declared a partner who works part-time for pay. Twelve (24%) had one or two children (aged 18 and under) living at home. Twenty-three (46%) had experienced shift work, and 40 (80%) had experienced working nights. Compared to other people their age, at baseline, the majority of participants described their physical health as either good (42%) or very good (34%) and their mental health as good (40%), very good (18%) or excellent (6%). Approximately one third reported their mental or physical health as either poor or fair.

### Perceptions of the impact of the SRRB

We report on the residents’ perceptions of the impact of the SRRB related to the senior residents’ wellness, ability to deliver quality health care, and medical education experience.

#### Wellness

After the intervention, residents’ perceptions of senior residents’ wellness improved for 1 of the 5 scales, worsened for 1 of the 5 scales, and remained unchanged for 3 of the 5 scales (Table 2). For the scale *allows exposure to personal harm*, the mean score decreased from 4.0 pre-intervention to 2.8 post-intervention, a decrease of 1.3 (95% CI -1.8 to -0.8; p < 0.001). For the scale *causes conflicting role demands*, the mean score increased from 2.9 pre-intervention to 3.7 post-intervention, an increase of 0.8 (95% CI 0.3 to 1.2; p = 0.002). There were no statistically significant differences in the mean scores for the scales *allows general wellness*, *allows healthy relationships*, and *causes feelings of isolation* (Table 2). Thus the residents in our study felt that the SRRB affected the seniors’ wellness in that the seniors were exposed to less personal harm at work and experienced more conflicting role demands than before the six month pilot, with no significant change in the other measures.

**Table 2:** Change in internal medicine residents’ perceptions of the impact of the schedule on aspects of the seniors’ self/work Pre- and post-intervention (N = 50)

**Aspects of senior resident self/work affected by 24 hour shifts**	**Pre**	**Post**	**Difference**	
**Senior resident wellness**	**Mean (SE)**	**Mean (SE)**	**Mean (95% CI)**	P value*
Allows general wellness	2.2 (0.1)	2.6 (0.1)	0.39 (-0.04 – 0.8)	0.07
Allows exposure to personal harm	4.0 (0.1)	2.8 (0.2)	−1.29 (-1.8 – -0.8)	<0.001
Causes conflicting role demands	2.9 (0.1)	3.7 (0.1)	0.77 (0.3 – 1.2)	0.002
Allows healthy relationships	2.6 (0.1)	2.7 (0.2)	0.05 (-0.4 – 0.5)	0.81
Causes feelings of isolation	2.3 (0.1)	1.9 (0.1)	−0.36 (-0.7 – 0.02)	0.06
**Ability to deliver quality health care**				
Allows potential for error	3.2 (0.1)	2.5 (0.1)	−0.68 (-1.1 – -0.3)	0.003
Allows clinical skills expertise	3.5 (0.1)	3.8 (0.1)	0.26 (-0.03 – 0.6)	0.07
Allows continuity of patient care	3.6 (0.1)	3.6 (0.1)	0.04 (-0.3 – 0.4)	0.80
Causes expenditure of emotional labour	2.2 (0.1)	2.2 (0.1)	0.07 (-0.2 – 0.3)	0.57
Allows work efficiency	3.4 (0.1)	3.6 (0.1)	0.21 (-0.1 – 0.5)	0.15
**Medical education experience**				
Allows successful teaching	3.0 (0.1)	3.5 (0.1)	0.48 (0.1 – 0.8)	0.0097
Allows medical skills proficiency	3.3 (0.1)	3.5 (0.1)	0.24 (-0.1 – 0.6)	0.14
Allows successful learning	3.1 (0.1)	3.5 (0.1)	0.42 (-0.02 – 0.9)	0.06
Allows staff physician supervision	3.1 (0.1)	2.7 (0.1)	−0.42 (-0.7 – -0.1)	0.0099
Causes rotation disruptions	4.1 (0.2)	3.3 (0.1)	−0.83 (-1.4 – -0.3)	0.003

*P value of <0.01 indicates a statistically significant difference between pre- and post-scores.

#### Ability to deliver quality health care

After the intervention, residents’ perceptions of senior residents’ ability to deliver quality health care improved for 1 of the 5 scales, and remained unchanged for 4 of the 5 scales (Table 2). For the scale *allows potential for error*, the mean score decreased from 3.2 pre-intervention to 2.5 post-intervention, a decrease of 0.7 (95% CI -1.1 to -0.3; p = 0.003). There were no statistically significant differences in the mean scores for the scales *allows clinical skills experience, allows continuity of patient care, causes expenditure of emotional labour*, and *allows work efficiency* (Table 2). Thus the residents in our study felt that the SRRB affected the seniors’ ability to deliver quality health care in that it allowed less potential for error than before the six month pilot, with no significant change in the other measures.

#### Medical education experience

After the intervention, residents’ perceptions of senior residents’ medical education experience improved for 2 of the 5 scales, worsened for 1 of the 5 scales and remained unchanged for the other two scales (Table 2). For the scale *allows successful teaching,* the mean score increased from 3.0 to 3.5, an increase of 0.5 (95% CI 0.1 to 0.8; p = 0.0097). For the scale *allows staff physician supervision*, the mean score decreased from 3.1 pre-intervention to 2.7 post-intervention, a decrease of 0.4 (95% CI -0.7 to -0.1; p = 0.0099). For the scale *causes rotation disruptions*, the mean score decreased from 4.1 pre-intervention to 3.3 post-intervention, a decrease of 0.8 (95% CI -1.4 to -0.3; p = 0.003). There were no statistically significant differences in the mean scores for the scales *allows medical skills proficiency* and *allows successful learning,* (Table 2). Thus the residents in our study felt that the SRRB affected the seniors’ medical education experience in that the seniors were better teachers and experienced fewer rotation disruptions, but received less staff physician supervision than before the six month pilot, with no significant change in the other measures.

## Discussion

The residents in our program felt that most aspects of the three domains of the senior resident’ wellness, ability to deliver quality health care, and medical education experience were unaffected by the introduction of the SRRB, but reported four improved perception shifts and two that had worsened. The SRRB’s combination of innovations, namely educational sessions on sleep hygiene, an electronic handover tool and a simulation-based medical education curriculum, may have successfully targeted some of the perceived potential negative consequences of duty hour restrictions and night float systems.

Study participants felt that the senior residents were exposed to less personal harm as a result of the SRRB. This is consistent with previous research showing that prolonged duty hours for residents increase their personal harm risk including needle stick injuries, motor vehicle collisions post-call, and burnout [1,6,9,18,19]. Study participants also felt that the SRRB caused increased conflicting role demands by reducing the senior residents’ ability to spend time with family, perform research, and trade work shifts compared to the traditional system of call. Despite this, there was no perceived change in senior residents’ general wellbeing. Previous studies have reported mixed results in residents’ health, wellness and quality of life after implementation of night float systems [9,12,14,30] with some reporting such improvements as more time available to study, as well as negative consequences, such as increased depression and feelings of isolation [20-22,36].

Benefits in patient care and outcomes have been previously reported after the implementation of duty-hour restrictions [3,9,10,12,14,16]. Our study results are consistent with these findings whereby participants felt that senior residents were less likely to commit a medical error with shorter duty hours. The resulting shorter shifts and increased number of patient care handovers between postgraduate trainees has been identified as a significant potential source of communication and medical errors [8,24,37-40]. Our participants did not feel there was a negative impact on continuity or quality of care provided by the senior resident as a result of the SRRB. The implementation of an electronic handover tool as part of the SRRB may have influenced these perceptions. A growing body of literature supports the use of such electronic tools in order to minimize this potential negative consequence [28,29].

The majority of research published has reported either no change or an improvement in the overall quality of medical education with the implementation of duty-hour restrictions [9,12,14,24,30]. Study participants felt that the senior residents’ educational experience was enhanced after the implementation of the SRRB with improved teaching effectiveness. This may be due in part to decreased senior resident fatigue during teaching duties given shorter consecutive duty hours and the addition of the simulation-based medical education curriculum. Both high-fidelity and low-fidelity procedural simulation have been successfully used as medical education tools in post-graduate training [31-34]. The study participants felt that the implementation of the SRRB allowed less staff physician supervision for the senior residents but did not feel there was any significant difference in the senior residents’ ability to learn successfully after the implementation of the SRRB. These perceptions may reflect a balance between the benefits of the simulation curriculum against the loss of both daytime medical education experiences and resident/staff physician handover contact, and direct supervision for night float residents. Participants felt there were fewer disruptions in other rotations, possibly reflecting that the SRRB eliminated the need to pull residents from other rotations to fulfill MTU call shifts, allowing residents on subspecialty rotations to complete their duties with fewer interruptions.

Our study should be interpreted in light of the study design. First, this study reports changes in internal medicine residents’ perceptions rather than objective outcomes. Perceptions are often important determinants of the ultimate success of such resident-driven structural and scheduling changes. Second, we chose to include all the residents in the study cohort. We recognize that we thus asked residents who did not personally experience aspects of the bundle as well as those who did to offer their perspective on how it may have affected the senior residents. We felt it was essential to include the perceptions of all residents as they were all stakeholders in this quality improvement initiative, and the rotation changes we examined could be permanently adopted at our institution. The junior residents’ perceptions of the impact of the SRRB on their more senior colleagues is important in the context of their reliance on the seniors as clinical mentors and teachers, and their forthcoming transition into the senior role. Third, the timing of a 6-month intervention during an academic year makes it very difficult to ensure that every resident has had the same experience at the start of the intervention. This is another reason that we chose to include all the residents as our cohort in this study, recognizing that even some of the post-graduate year 2 senior residents would have had limited experience with the pre- and possibly post-intervention rotation schedule. Accordingly we acknowledge that at the time of initiation of the study pilot, the surveyed participants would have had varied rotations and clinical experiences. Fourth, this study was performed at a single institution. While the response rates were very high the generalizability of these study results may be limited as each individual institution is subject to its own culture and practice. We were limited by the size of our program and therefore our study may have been insufficiently powered to yield other statistically significant differences. Lastly, every institution and postgraduate training programs is a dynamic entity, and the results reported may have been influenced by changes within our site and residency program during the study period.

## Conclusions

The SRRB model is unique in that it integrates an education module on sleep hygiene, an innovative electronic handover tool and a simulation-based medical education curriculum into a night float system, potentially easing some of the negative consequences of duty hour restrictions. The results of this study have helped the integration of the SRRB into current practice at our institution. The study results were widely disseminated to the medical educators, internal medicine residents and staff physicians, facilitating acceptance of this major change to the residents’ schedule and work life. Since the pilot, we have used feedback to guide ongoing improvements to the SRRB in an iterative fashion. The number of consecutive weeknights of call has been reduced to 4, and 3-night weekend coverage has been added. A similar schedule change is planned for the junior residents this coming academic year. The simulation curriculum, in part because of its successful inclusion in the SRRB, has enjoyed a rapid expansion, with more preceptors involved as procedural task trainers and the creation of additional high fidelity test cases. The patient care handover communication tool is firmly integrated into the standard of care on the MTUs, and anecdotally, the MTU trained residents continue to use it elsewhere in the hospital. Future study on this topic should include objective measures of patient outcomes, patient safety, resident health and safety, and medical education experience in order to validate the perceptions of our study participants. In addition, we could expand our study to include perceptions of other members of the MTU multi-disciplinary care team such as attending physicians, nurses, occupational therapists and pharmacists.

## Competing interests

The authors declare that they have no competing interests.

## Authors’ contributions

GF contributed to the study concept and design, interpretation of data, drafting, and revision of the article. ME contributed to the study concept and design, interpretation of data, and revision of the article. SK contributed to the study concept and design, interpretation of data, and revision of the article. EM contributed to the study concept and design, interpretation of data, and revision of the article. JEW contributed to the study concept and design, analysis and interpretation of data, revision of the article. JdG contributed to the acquisition and analysis of data, interpretation of data, and revision of the article. AL contributed to the analysis of data, interpretation of data, drafting and revision of the article. GB contributed to the study design, acquisition of data, interpretation of data, and revision of the article. AB contributed to the study design, interpretation of data, and revision of the article. JG contributed to the study design, interpretation of data, and revision of the article. JBL contributed to the study concept and design, analysis and interpretation of data, drafting and revision of the article. All authors read and approved the final manuscript.

## Authors’ information

Jane B Lemaire: Senior investigator.

## Pre-publication history

The pre-publication history for this paper can be accessed here:

http://www.biomedcentral.com/1472-6920/13/115/prepub
